# Isolated Bacteria from the Uteri of Camels with Different Reproductive Backgrounds: A Study on Sampling Methodology, Prevalence, and Clinical Significance

**DOI:** 10.3390/vetsci10010039

**Published:** 2023-01-05

**Authors:** Behnam Asadi, Fahimeh Seyedasgari, Iradj Ashrafi Tamai, Mehdi Yarmohammadi, Reza Ebadi, Ellen Kim, Abbas Barin

**Affiliations:** 1 Camel Advanced Reproductive Technologies Center, Zabeel Office, Government of Dubai, Dubai P.O. Box 5928, United Arab Emirates; 2 Department of Microbiology, Faculty of Veterinary Medicine, University of Tehran, Tehran P.O. Box 1417935840, Iran

**Keywords:** bacteria, microbiota, uterine discharge, pregnancy

## Abstract

**Simple Summary:**

The mammalian uterus has been shown to host a range of microorganisms with or without potential pathogenic capacity. In camels, microorganisms from infertile subjects have been isolated using various sampling methodologies, but not much is known about the range of expected microbes that can be harbored in the uteri of camels without a complicated reproductive performance. The current results indicated up to 66% of camels yield bacteria from uterine samples with a higher frequency among sub-fertile subjects; yet, uterine bacterial burden does not necessarily indicate uterine pathology in otherwise reproductively asymptomatic animals. The application of small-volume lavage for uterine sampling might change our understanding about the range of uterine isolates in camels.

**Abstract:**

The objectives of this study were to comparatively identify the common bacterial isolates from the uteri of camels coming from different reproductive backgrounds after standardizing the sampling method and to investigate the association of clinically measurable parameters with uterine colonization by these isolates. The uterine samples from 856 dromedary camels yielded a total of 17 different bacterial species with a higher proportion of sub-fertile camel uteri being colonized by bacteria (66.6%) as compared to nulliparous, recently calved, and those with unknown reproductive history combined (44.2%; *p* < 0.05). Camels with body condition scoring < 3 and those with a consistently echogenic appearance of the uterine lumen by sonography were more likely to be positive on uterine culture, while the presence of pus in uterine discharge was not associated with the odds of bacterial isolation (*p* > 0.05). While certain strains were more likely to be obtained from the uteri of the sub-fertile group (*p* < 0.05), embryo transfer to camels with a positive uterine culture in the absence of other gross reproductive pathologies did not necessarily affect the overall pregnancy rate compared to recipients with a negative uterine culture (*p* > 0.05). In conclusion, a relatively high bacterial load can be identified from the uteri of both sub-fertile and normal dromedary camels, with a higher frequency among the former. The uterine ultrasonography and evaluation of the body condition score can help in identifying the camels in which uterus is contaminated by bacteria.

## 1. Introduction

Different parts of the reproductive tract in mammals have been shown to harbor their own distinct microbiota and a wide variety of opportunistic and pathogenic bacteria [1]. In large animals, uteri can potentially be contaminated with considerable loads of microorganisms during late gestation and throughout the postpartum period, and the respective phyla can fluctuate during the following periods and at different times of the estrous cycle [2,3]. The contaminating bacteria may or may not underpin uterine infections depending mainly on the type and number of the bacteria, presence of virulence factors and the host response to contamination [4,5,6] and should infection develop, they could eventually lead to significant reproductive losses by adversely affecting the follicular growth [7,8], conception rate and embryo survival [9,10] and disturbing normal hormonal profile of the affected animals [11].

Similar to other large farm animals, numerous microorganisms have been isolated from the uteri of dromedary camels with a preponderance of bacteria [12]. Isolation rates are different among reports and range between 28% [13] to 92.5% [14] of the cultured samples. However, the majority of attempts at uterine culture in dromedaries have focused on infertile camels and abattoir samples carried out with varying methodologies [13,15,16,17].

In dromedary camel, uterine infections present the most frequent diagnosis among repeat breeders with clinical endometritis caused by bacteria comprising up to 32.2% of all reproductive referrals in barren females [17,18]. Despite the consensus on the role of bacteria in instigating uterine infections in infertile camels, no study has investigated the common isolates from the camels without a problematic reproductive background. Data does not converge on the frequency and type of microbial isolates, and little is known about the respective clinical significance of these isolates. To address these gaps, the current study was conducted to comparatively investigate the common bacterial isolates from camel uteri with different reproductive backgrounds. Subfertile camels, nulliparous, recently calved and those of unknown reproductive background managed under the current breeding practices were sampled after standardizing a sampling method to ascertain the type and frequency of uterine isolates in this species. We aimed to investigate the links between clinically measurable parameters and the possibility of uterus colonization by microorganisms. 

## 2. Materials and Methods

### 2.1. Experimental Design

Two sets of prospective observational experiments were carried out during camel breeding seasons in 2017–2020. In Experiment 1, a total of 72 subfertile camels were randomly sampled for uterine culture both by swab and by the small-volume lavage technique (SVL). The objectives included investigating the prevalence of positive uteri and the type and frequency of bacterial isolates, as well as comparative evaluation of uterine sampling methods to standardize a reliable technique that facilitates more accurate identification of the significant uterine isolates while minimizing false results. In Experiment 2, a total of 784 uterine samples from camels were obtained to investigate the prevalence of uterine colonization by bacteria in camels with different reproductive histories. For this experiment, nulliparous camels (n = 44), calved camels (n = 264) and those with unknown reproductive performance (n = 476) were sampled by SVL and the frequency and the type of isolates were investigated across the groups and compared to the subfertile camels. The association between isolation of bacteria and the presence of uterine discharges, body condition of the subjects, age, cyclicity status, and the appearance of the uterus on ultrasonography scanning was investigated by a logistic regression model. In Experiment 3, the clinical significance of bacterial colonization of the uterus was investigated retrospectively. For this purpose, two groups of camels with a positive uterine culture in the absence of gross reproductive pathologies were used as embryo recipients, and the obtained pregnancy rates were compared to a cohort of camels with a negative uterine culture. The first group consisted of camels treated for a proven record of subfertility prior to transfer, while the second group did not indicate a complicated background and was not treated.

### 2.2. Animals

The examinations were conducted at the Camel Advanced Reproductive Technologies Center (CARTC), Dubai, UAE, during breeding season 2017–2020. Animals were referred for the purpose of preparation for natural cover or serving in embryo transfer plans as donors or recipients. All camels tested negative for brucellosis, were 6 to 15 years old and ranged from 1.5 to 5 in a 1-to-5-scale body-condition scoring system described elsewhere [19]. The majority of animals were placed in open shade and fed mainly on Rhodes grass and alfalfa hay with ad libitum access to water. Camels were further sub-grouped with regard to a verified history, their reproductive performance during the experiment and the reproductive examinations. A subfertile subgroup (n = 72) consisted of multiparous animals that remained barren after at least 3 controlled mating sessions with bulls of proven fertility or those who failed to become pregnant after a minimum 3 rounds of embryo transfer in the absence of gross reproductive pathologies. Nulliparous camels included in the study were selected with regard to history, aging < 7 years old with small-sized genitalia in rectal palpation and characterized by the presence of hymen (n = 44). A recently calved group consisted of camels with a verified history of parturition between 6–9 months (n = 264). The third group consisted of multiparous camels with uncertain previous reproductive performance (n = 476). 

### 2.3. Reproductive Examinations

**Figure 1:**
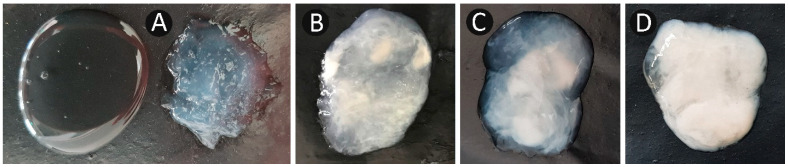
(**A**–**D**) Uterine discharges from female dromedary camels; (**A**) clear/mucoid gelatinous discharge without pus (right) and translucent rectal examination gel (left); (**B**) mucoid discharge with specks of pus < 25% (PGD1); (**C**) purulent discharge with 25–75% pus (PGD2); (**D**) purulent discharge with >75% pus (PGD3).

Camels were restrained in a standing position and subjected to a thorough reproductive examination by standard transrectal palpation and by an ultrasonography instrument equipped with a 9 MHz linear-array transducer (Aloka^®^, Hitachi, Tokyo, Japan). Ovaries were examined for incidence of hydro-bursitis, and cyclicity status was categorized based on the presence or absence of follicular activity on two consecutive exams. Uteri were examined for consistency, presence of severe adhesions in the utero-ovarian bursa and signs of fluid accumulation within the lumen. Examined uteri were categorized with regards to the echogenic reflection in the uterine lumen into three categories: 1- normal echogenicity (NE) showing minimum echogenic reflection along the lumen, 2-mild echogenicity (ME) showing mild echogenic spots or intermittent echogenic line along the uterine lumen, and 3-consistently echogenic (CE) showing continuous thick echogenic line or pockets of fluid along the uterine lumen. For vaginal examination, the tail was wrapped, and the perineal area was thoroughly washed with chlorhexidine soap 4% and cleansed. A sterile vaginal speculum was then inserted to the vagina, and the cervical opening was inspected for bruises and injuries. Discharges originating from the uterus were categorized into three groups, as described elsewhere, with modifications [20]: 1-no discharge (ND): absence of any discharge on cervical opening and in the vaginal space; 2-gelatinous discharge (GD): clear to cloudy mucus with no pus; 3-purulent discharge (PD): containing pus in the mucus. Purulent discharges were further categorized as grade 1 (PDG1; mucoid discharge with pus), grade 2 (PDG2; mucopurulent discharge containing 25–75% pus) and grade 3 (>75% pus) (Figure 1A–D).

### 2.4. Uterine Sampling

Uterine samples were collected by swab [21] or small-volume lavage technique [22]. Briefly, a double-guarded swab (Equivet^®^, Langeskov, Denmark) was guided through a sterile vaginal speculum towards the outer opening of the cervix. The swab was pushed out of the protecting guards at two steps and directed towards the base of both uterine horns by rectal manipulations, wherein the swab was gently rotated against the surface of the lumen for 30 s before being retracted to the protecting guards. For the small-volume lavage technique, a sterilized uterine flushing catheter (Minitube^®^, Munich, Germany) was carefully passed towards the cranial vagina through the sterilized vaginal speculum. To further minimize possible contaminations, a sterile plastic cover was applied to protect the catheter during the passage through the vaginal space. The plastic cover was torn, and the tip was then located in the outer opening of the cervix and further guided by rectal manipulations towards the body of the uterus, where the cuff was inflated to fixate the catheter. A total of 60 mL of sterile pre-warmed Ringer lactate solution was then infused into the uterus through the catheter, followed by gentle massaging of the horns for one minute. The recovered fluid was then collected in a sterile syringe. Samples were placed in a cool box and immediately transferred to the laboratory.

### 2.5. Uterine Culture

SVL content was centrifuged at 1500× *g* for 15 min in a refrigerated centrifuge, and the supernatant was decanted. The precipitant was re-suspended in 1 mL PBS and used for culture. Obtained swabs and the SVL were inoculated onto MacConkey agar and blood agar containing 5% citrated sheep-blood plates (Merck Co., Darmstadt, Germany) and incubated aerobically and anaerobically for 24 to 48 h at 37 °C. The result was considered a single substantial growth in the primary culture if >95% of the grown colonies indicated phenotypic similarity. Colonies were visually inspected for morphology and motility and were further subjected to microbiological tests, as well as standard biochemical identification of the isolates as described elsewhere [23]. Samples were inoculated onto Sabouraud Dextrose agar (Merck Co., Darmstadt, Germany) supplemented with antibiotic (chloramphenicol; 0.05 g/L) and kept at 30 °C for 7–10 days before being considered negative [24]. For campylobacter isolation, samples were inoculated in charcoal differential agar (CCDA) plate and incubated in micro-aerobic conditions (Co2 8%) at 42 °C for 24–48 h. Colonies suspected of being campylobacter species were examined for cell morphology by optical microscope [25].

### 2.6. Uterine Treatments

Majority of the camels with a positive uterine culture for a major pathogen, which also indicated a proven history of subfertility, were subjected to uterine flushing with 1 L of prewarmed Ringer Lactate solution, followed by intramuscular administration of 2 mL ecbolic medicine (Oxitocina^®^, Over, Santa Fe, Argentina) and received intrauterine infusion of either 500 mg Cephapirin (Metricure^®^, MSD, Rahway, NJ, USA) or a combination of 200 mg gentamicin and 600 mg cefalexin monohydrate (Metrisan^®^, Over, Santa Fe, Argentina) or 900 mg cefquinome sulfate (Metrisafe^®^, Afarin darou, Tehran, Iran) on the following day based on the results of microbial sensitivity test.

### 2.7. Superovulation and Embryo Transfer

All embryo transfer procedures were carried out during the breeding season from November to May by one expert as described elsewhere with modifications [26]. In brief, donors were super-ovulated with a total of 400 NIU of FSH (Folltropin^®^, Vetoquinol, Lure, France) in decreasing doses, 12 h apart over a six-day period (4-4, 2.5-2.5, 1.5-1.5, 1-1, 0.5-0.5, 0.5-0.5 mL), and referred for mating 36 h after the last injection. Embryos were recovered 8.5 days after the mating by non-surgical uterine flushing and non-surgically transferred to recipients’ uterine horn on day 7.5 after induction of ovulation. Embryos were evaluated and categorized with regard to the grade (transferable and nontransferable) and shape (spherical and collapsed) [27,28]. The pregnancy data set included the recipients of transferable embryos with spherical shapes, and pregnancy was confirmed 20 ± 3 days after transfer by observing embryonic fluids in the presence of a viable CL together with embryonic proper or heart beat in ultrasonographic scanning of the uterus. 

### 2.8. Statistical Analysis

The proportion of camels with bacterial burden after culture was calculated as the number of camels with a positive uterine culture in each group divided by the total number of camels in the same group. The isolation frequency of a given bacteria was presented as the proportion of the isolated colony relative to all isolated colonies while prevalence of each isolate was calculated as the total number of the respective isolate to all camels in the same group. The frequency distribution of data was compared by the chi-square test of independence and further investigated by 2 × 2 contingency tables with odds ratios calculated when required. Fisher’s exact test was used when the number of observations per cell was less than five. The association between bacterial isolation from the uteri of the subjects with the type of animal (subfertile vs. uncomplicated reproductive history), body condition score (<3 vs. ≥3), age group (<10 years vs. >10 years), cyclicity (cycling vs. non-cycling), type of uterine discharge (non-purulent vs. purulent) and findings of uterine scanning (presence vs. lack of consistent echogenic line within the lumen), and the interactions was investigated by a logistic regression model within the generalized linear model function [29]. The association between isolation of bacteria from the uteri with pregnancy rates after embryo transfer was investigated by Cochran–Mantel–Haenszel extension of the chi-square test controlling for the effect of the breeding season. All statical procedures were performed by SPSS version 22 (IBM, Armonk, NY, USA).

## 3. Results

A total of 886 female dromedary camels were sampled in Experiments 1 and 2. After culture, five plates were discarded due to heavy contamination, possibly during the handling procedure. Fungi growth was observed from seven samples, all in conjunction with bacterial growth. A total of 13 camels were afflicted by one-sided and two-sided ovarian hydro-bursitis (n = 9 and n = 4, respectively), of which six camels were positive on uterine culture (46.1%). Pyometra was observed in five cases, characterized by accumulation of turbid fluid in the uterus together with the presence of a viable CL. These samples were excluded from the experiment.

### 3.1. Experiment 1

Table 1 summarizes the isolates from the samples taken by small-volume lavage (SVL) and swab technique. Camels with a confirmed history of subfertility indicated a relatively high rate of bacterial isolation from their uteri, which did not significantly vary between sampling methods (66.6 vs. 61.1; *p* > 0.05). A total of 88 isolates were obtained from SVL samples compared to 74 with the swab technique. The isolation frequency of organisms (isolate/total isolates) did not differ significantly between methods, but there was a notable trend for better isolation of Gram-negative bacteria by SVL (OR = 1.78, CI [0.95–3.33]; *p* = 0.07).

### 3.2. Experiment 2

**Figure 2:**
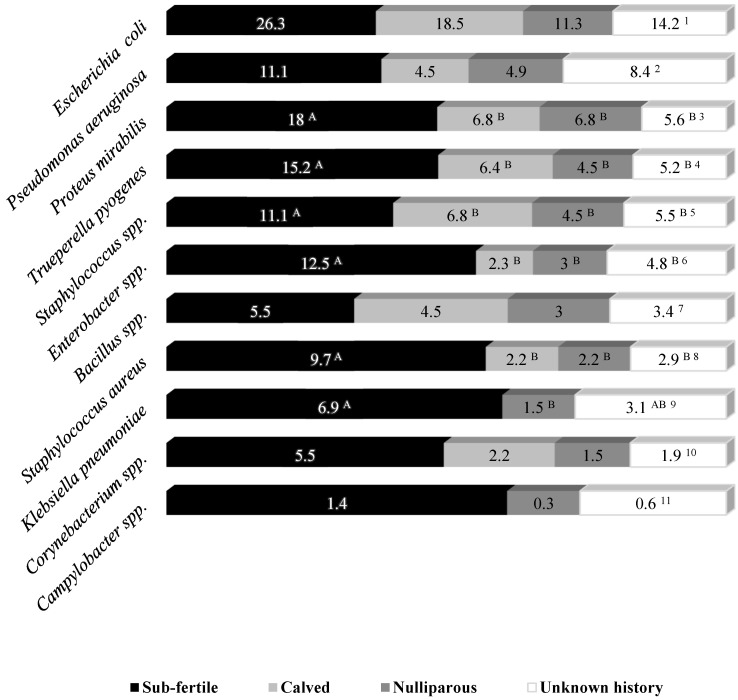
Comparative prevalence of major uterine bacterial isolates in subfertile camels, calved camels, nulliparous and those with unknown reproductive history. Figures represent percentage of isolates in each category (isolate/total number of camels in the same category). Different capital superscripts indicate significant differences in the frequency distribution among the four groups (*p* < 0.05). (1) *p* = 0.120; (2) *p* = 0.224; (3) *p* < 0.008; (4) *p* < 0.031; (5) *p* = 0.217; (6) *p* = 0.051; (7) *p* = 0.767; (8) *p* < 0.024; (9) *p* = 0.029; (10) *p* < 0.089; (11) *p* < 0.999.

A total of 784 uterine lavage samples were obtained from camels in Experiment 2. The type and the isolation frequency of bacteria from the uteri of different groups of camels are presented in Table 2. Overall, a higher number of subfertile camels tested positive for uterine culture as compared to nulliparous, recently calved and multiparous camels with unknown history combined (Table 2; *p* < 0.05). In camels that tested positive, overall isolation frequency and the frequency of each isolate did not differ (*p* > 0.05; Table 2). The overall prevalence of uterine isolates among the whole population of subfertile camels was also higher compared to other three types of camels (*p* < 0.05). Similarly, there was a higher number of each isolate obtained from their uteri with a significant difference in the proportion of *Proteus mirabilis, Trueperella pyogenes, Staphylococcus* spp. and *Klebsiella pneumoniae* compared to the rest of the groups (*p* < 0.05; Figure 2).

Table 3 presents the data composition of the clinical features in experimental subjects and respective bacterial isolation from their uterus. Frequency of positive uteri did not differ among camels with different age groups and body condition scoring (*p* > 0.05). However, a higher proportion of bacteria were obtained from subfertile camels, the camels with no discharge from uterus compared to those with purulent discharges and those with the presence of a continuous echogenic line within the lumen during ultrasonographic evaluation of the uterus (Table 3; *p* < 0.05).

**Figure 3:**
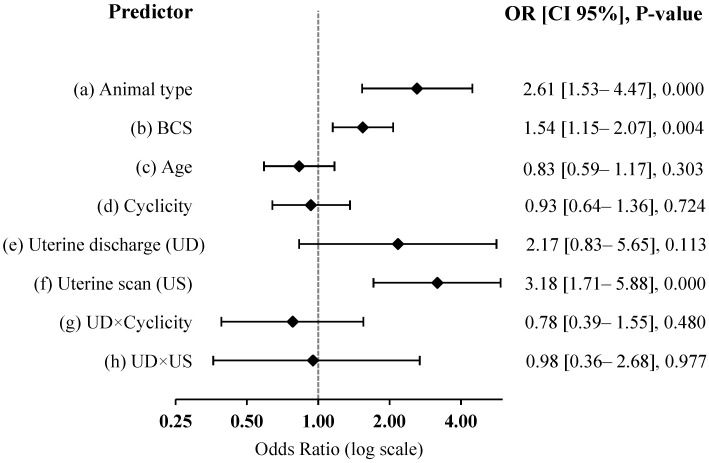
Graphical presentation of the association between clinical measures and the odds of bacterial isolation from the uterus in dromedary camel. (a) subfertile vs. calved and& unknown; (b) BCS < 3 vs. BCS ≥ 3; (c) Age ≤ 10 y vs. Age > 10 y; (d) active ovary (AO) vs. inactive ovary (IO); (e) no discharge and gelatinous discharge (ND) vs. purulent discharge (PD); (f) consistently echogenic appearance (CE) vs. non-echogenic and mild-echogenic appearance (NE); (g) ND × AO vs. PD × IO; (h) ND × CE vs. PD × NE.

Figure 3 depicts the extent of association between the observed clinical features in experimental subjects with the likelihood of bacterial isolation from their uteri. The results indicated a significant association of animal type, BCS and ultrasonography findings with uterine contamination (*p* < 0.05). Subfertility, BCS < 3 and presence of a consistent echogenic line in the lumen increased the likelihood of bacterial isolation (*p* < 0.05) while camels’ cyclicity as indicated by presence or absence of follicular activity, camel age (<10 years or >10 years) and the presence of purulent uterine discharges were not associated with the odds of bacterial isolation (*p* > 0.05; Figure 3).

### 3.3. Experiment 3

**Figure 4:**
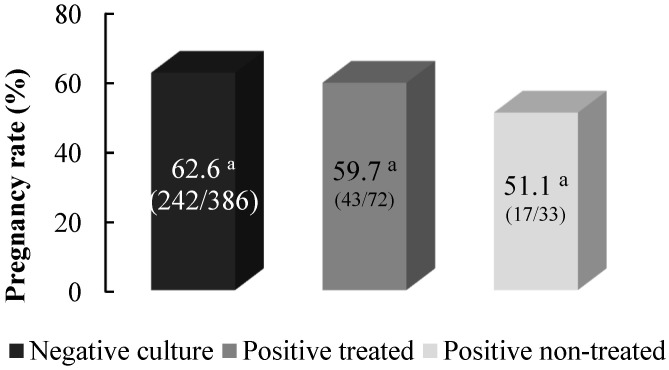
Pregnancy rates (%) after embryo transfer in camels with a negative and positive uterine culture. Different superscripts indicate significant difference in the frequency distribution of subjects among groups (*p* < 0.05).

Figure 4 presents the comparative pregnancy rates among camels with negative and positive uterine cultures after embryo transfer. The pregnancy data set was selected from the records of 752 embryo transfer procedures carried out during the same timeframe of the experiment. The inclusion criteria were transfer of a single transferable embryo with spherical shape to an otherwise clinically healthy recipient. Overall pregnancy rates after embryo transfer did not differ among subfertile camels treated after a positive uterine culture and non-treated camels with a positive uterine culture in the absence of gross reproductive signs compared to a cohort of camels with a negative uterine culture (*p* > 0.05; Figure 3). 

## 4. Discussion

The current study investigated the common microorganisms frequently isolated from the uteri of dromedary camels, which are typically referred to clinicians for reproductive procedures, such as preparation for natural cover or serving in embryo transfer programs as donors or recipients. To the best of our knowledge, this is the first study to comparatively report the range of bacteria that are recovered from the uteri of sub-fertile camels and those without a complicated reproductive background. 

Results indicated that various species of microorganisms can be recovered from the uteri of dromedary camels (18 species) with the absolute preponderance of bacteria (17 species) (Table 1 and Table 2). Regardless of the reproductive background, a relatively high proportion of camels can be positive on uterine culture (34.1 to 66.6%). Studies on cattle and equine have documented the presence of a dynamic uterine microbiota that contaminate the uterus during prepartum and immediate postpartum periods [1,3]. In camels, the nature of the mating procedure is that it occurs in sitting position and involves intrusion of penis deep into the uterus, together with the common practice of breeding typically involving repeated referrals for mating, resulting in multiple insults to the reproductive tract and predisposes the uterus to a high contamination burden [12]. An interesting observation in the current study was that 34.1% of nulliparous camels managed under common breeding practices also yielded a considerable load of bacteria from their uterine samples (Table 2). 

Despite the relatively high prevalence of camels with uteri colonized by bacteria, embryo transfer into multiparous recipients with a positive uterine culture that were otherwise reproductively sound did not lower the overall pregnancy rate to a significant level when compared to those camels with a negative uterine culture (Figure 4), corroborating the fact that a considerable proportion of camels with bacterial burden in the uterus do not develop related uterine pathologies that prevent normal conception. Metagenomic analysis of uterine samples in dairy cattle, as an animal model in which uterine infection is extensively studied, revealed that cows afflicted with metritis or those maintaining a healthy uterus had a similar uterine microbiota after parturition [30]. The development of infection subsequent to contamination have been proposed to mainly depend on individual animal resilience against infection in terms of immunity functions that aim to counteract pathogens by killing and removing them [5,20]. Between-strain interaction is another significant factor in this regard since research has revealed the association between the presence of coagulase-negative *staphylococci* and *α-hemolytic* with a reduced risk of endometritis [31], while some pathogens, such as *T. pyogenes,* were shown to persist in the contaminated uterus and to act synergistically with other strains to enhance the severity of uterine pathologies [6,32]. Possible interactions between isolated organisms from the uterus and the dynamism between contamination and infection in camels requires further investigations.

Prevalence of uteri with a positive bacterial culture and the isolation frequency of different strains was higher among subjects with a recorded history of subfertility compared to camels with uncomplicated or unknown previous reproductive performance (*p* < 0.05, Table 2). This finding highlights the significance of bacterial involvement in lowering reproductive performance in camels and is in-line with a previous study in which the total microbial recovery rate from 152 uteri obtained from the abattoir was 23.6%, while 81.6% of camels with active uterine infection indicated by cytology yielded bacteria in their samples [13]. The most frequent isolates were *E. coli* (16.4%), *Enterobacter cloacae* (12.8%), *Klebsiella pneumoniae* (4.2%) and *S. aureus* (2.8%) [13]. In another study on barren camels, 86.6% of repeat breeder subjects (39/45 camels) had positive uterine cultures yielding 12 different bacterial strains, out of which *Trueperella pyogenes, Staphylococcus aureus,* and *Streptococcus pyogenes* consisted of 46.1% of all isolated organisms (14, 13 and 9 of all 78 isolates) [17]. 

Comparative data on the range of uterine isolates that affect camels other than those suffering from fertility problems are scarce to non-existent. In the current study, the proportion of isolated bacterial strains was not different among positive uteri of camels regardless of their reproductive background (Table 2). However, the prevalence of *Proteus mirabilis, Trueperella pyogenes, Staphylococcus aureus, Enterobacter aerogenes* and *Klebsiella pneumoniae* within the population of subfertile camels was higher compared to the rest (Figure 2). From an epidemiological point of view, this might indicate a more serious involvement of certain strains over others in instigating uterine pathologies subsequent to contamination of the uterus. Significantly higher relative abundance of Bacteroidetes spp. and fusobacterium spp. in cows with clinical metritis compared to healthy counterparts has been proposed to reflect their importance in development of uterine infection in dairy cows [30]. Future studies that incorporate a gold-standard method into their design for diagnosis of ongoing inflammation should investigate the relative clinical significance of different isolates in camels.

The variations in isolation rates among reports might partly be attributed to the sampling methodologies applied in different studies, which could lead to raised false-negative or false-positive results. In the first experiment, the use of small-volume lavage technique for uterine sampling resulted in isolation of more bacteria after culture compared to sampling by standard double-guarded swab technique (88 vs. 74 isolates from 72 camels; Table 1). The absolute number of isolates was higher for almost all major pathogens (Table 1), probably due to facilitating a complete sampling of the whole endometrial surface through this technique [33]. This is particularly important for *Klebsiella pneumoniae*, which the swab technique failed to detect (Table 1). A classic study on normal and aged subfertile mares reported similar observations, in which, from 51 sampled cases, SVL yielded 20 pathogenic organism colonies compared to only 4 colonies by swab method (*p* < 0.05) [22]. Authors reported a lack of agreement in the culture score of pathogenic microorganisms from normal mares between SVL and swab (r = 0.20, *p* > 0.05) and found the former to be more accurate with regards to cytologic evidence of inflammation in all mares [22]. Sampling by SVL seems to favor more accurate isolation of almost all strains and Gram-negative bacteria in particular (OR = 1.78, *p* = 0.07; Table 1). Works on equines also indicated a more efficient detection of Gram-negative bacteria and *E. coli* in particular when samples were taken by SVL, leading to a rise in the diagnostic sensitivity of uterine infection by culture from 34% to 71% [34]. The current results suggest future clinical studies that aim to standardize a diagnostic approach for the detection of uterine infection in camels would potentially benefit from application of this technique as well. 

In camels, catarrhal, mucopurulent or purulent vaginal discharge is generally regarded as clinical signs of uterine infection [12,35]. The same feature forms the hallmark of diagnosis for clinical endometritis in cattle [8,11] with the presence of pus discharge, whether found on external inspection or by vaginoscopy being associated with impaired fertility [10]. Our result indicated that camels with purulent discharge from uterus did not necessarily indicate a higher risk of being positive on uterine culture compared to those camels with no discharge or with clear discharge (*p* > 0.05, Table 3 and Figure 3). This does not refute the possibility of the same camels going through an inflammatory reaction due to a possible infection since presence of numerous neutrophils was confirmed by microscopic evaluation of the purulent discharges (data not shown). A previous observation in our laboratory was that 28.8% of the referred camels showed the presence of purulent discharge from uterus at their first examination, while another 30.2% were diagnosed with the condition later in the season, with a tendency to have more pus in their discharge (grade 2 and 3) in the latter group (*p* = 0.023) [36]. It can be hypothesized that camels with purulent discharge might be recovering from a prior encounter with microorganisms and camels with bacterial load in the absence of discharges may develop an immune response to contamination/infection at a later stage. Alternatively, it is possible to think that purulent vaginal discharge may represent a different manifestation of reproductive tract disease [37], since up to 38% of animals with purulent vaginal discharges did not indicate cytological evidence of uterine inflammation [38]. 

On the other hand, lower BCS and the presence of echogenic material inside the lumen were associated with a higher likelihood of a positive uterine culture (*p* < 0.05), while ovarian cyclicity and age group of animals were not associated with that endpoint (*p* > 0.05; Table 3 and Figure 3). In agreement with this observation in camels, studies on other large animals have showed that underlying inadequate nutrition lowering BCS can facilitate entry and colonization of the uterus by microorganisms through altering normal anatomical structures that act as physical barriers to contamination [39,40] or triggering suppressed immune functions required to maintain the microbial hemostasis of uterus through negative energy and protein balance [41,42]. A consistently echogenic line in longitudinal section of the uterus might represent the presence of pus, which has been proposed to characterize clinical endometritis at 21 days postpartum onwards without related discharge in the vagina of dairy cows [38]. Likewise, in the current study, the nature of contents in the uterus observed by sonography seemed to differ from uterine discharges observed during vaginoscopy examination with regard to the insignificant interaction between the two (Figure 3).

It is noteworthy to mention that, unlike bacteria, molds constituted rare isolates from the sampled uteri with only seven positive plates, all in conjunction with growing bacteria, while none belonged to the sub-fertile group. A previous study on fungal flora of the dromedary reproductive tract reported isolation of various Candida spp. from vestibule and vagina, cervix and uterine body but not uterine horns in 40 out of 50 sampled animals [24]. *Candida albicans* constituted 0.4% of all isolates from 156 pathologic uteri, signifying the relatively low importance of molds [13], while another report found this organism to be the second most cultured isolate from the uteri of camels suffering from subclinical endometritis [43].

Bacterial strains, such as *Mannheimia haemolytica, Salmonella* spp., *Listeria monocytogenes* and *Pasteurella multocida*, which did not comprise a considerable proportion of isolates might bare less clinical significance and have probably reached the uterus due to a previous or ongoing systemic illness, since at least 2 respective camels were known to have had a history of a chronic alimentary situation caused by *Salmonella* spp. However, *Campylobacter* spp., which are known reproductive pathogens in camels [18], should be considered more seriously despite the low observed prevalence of 1.1% (Table 2). This latter organism might have gone undetected in recent studies due to the inaccuracy of sampling methods. 

## 5. Conclusions

The current results shed light on the range of expected uterine bacteria when dealing with camels from different reproductive backgrounds and provides preliminary evidence for the presence of uterine microbiota in this species while putting some strains above others with regard to a potential clinical significance. To clarify the interaction of the colonizing bacteria of the uterus with the eventuality of uterine infection in camel, future longitudinal experiments should investigate the interrelationships among these strains with regard to the reproductive timeline of camels and interpret the culture results by incorporating a gold-standard method (histopathology or cytology) for detection of inflammation into their design. Such experiments will benefit from a standard SVL sampling methodology for more accurate culture results. Moreover, under practical conditions with limited access to accurate history or laboratory facilities, body condition scoring of camels and ultrasonographic evaluation of the uterus will help to identify the camels at a greater risk of being contaminated with uterine bacteria.

## Figures and Tables

**Table 1:** Bacterial isolates from the uteri of subfertile camels obtained by different sampling methods.

	Sampling Method (n = 72)	
	Lavage	Swab
Total positive uteri (%)	48 (66.6)	44 (61.1)
Total isolates	88	74
Discarded plates	-	2
Gram −/+ ^1^	53/35	34/40
Organism (%)		
*E. coli*	19 (21.5)	11 (15.0)
*Proteus mirabilis*	13 (14.7)	9(12.3)
*Trueperella pyogenes*	11 (12.5)	10 (13.6)
*Pseudomonas aeruginosa*	8 (9.1)	5 (6.8)
*Enterobacter aerogenes*	8 (9.1)	9 (12.1)
*Staphylococcus* spp.	8(9.1)	11 (15.0)
*Staphylococcus aureus*	7 (7.9)	7 (9.6)
*Bacillus* spp.	4 (4.5)	5 (6.8)
*Klebsiella pneumoniae*	5 (5.7)	-
*Corynebacterium* spp.	4 (4.5)	4 (5.4)
*Campylobacter* spp.	1 (1.1)	1 (1.3)
*Streptococcus* spp.	-	1 (1.3)
*Citrobacter freundii*	-	1 (1.3)
*Fungi*	-	-

^1^-OR= 1.78, CI [0.95–3.33], *p* = 0.07.

**Table 2:** Uterine bacterial isolates from camels with different reproductive history.

	Reproductive History (n = 856)				*p*-Value
	Sub-Fertile (n = 72)	Nulliparous (n = 44)	Calved (n = 264)	Unknown (n = 476)	
Total infected uteri (%) (n = 394)	48 (66.6)	15 (34.1)	112 (42.4)	219 (46.0)	0.136
Groups combined (%)	48 (66.6) ^a^	346/784 (44.2) ^b^			0.035
Total isolates (%) (n = 556)	88	21	156	291	-
Isolates/Group	1.22 ^a^	0.48 ^b^	0.59 ^b^	0.61 ^b^	<0.002
Isolates/infected uteri	1.83	1.4	1.39	1.32	0.457
Isolates (%) ^1^					
*E. coli (n = 141)*	19 (21.6)	5 (23.8)	49(31.4)	68 (23.3)	0.474
*Pseudomonas aeruginosa (n = 63)*	8 (9.1)	2 (8.7)	13 (8.3)	40 (13.7)	0.411
*Proteus mirabilis (n = 61)*	13 (14.7)	3 (14.3)	18 (11.5)	27 (9.3)	0.586
*Trueperella pyogenes (n = 55)*	11 (12.5)	2 (8.7)	17 (10.9)	25 (8.6)	0.766
*Staphylococcus* spp. *(n = 47)*	8 (9.1)	3 (13.0)	12 (7.7)	24 (8.2)	0.827
*Enterobacter aerogenes (n = 40)*	8 (9.1)	1 (4.3)	8 (5.1)	23 (7.9)	0.650
*Bacillus* spp. *(n = 30)*	4 (4.5)	2 98.7)	8 (5.1)	16 (5.5)	0.862
*Staphylococcus aureus (n = 28)*	7 (7.9)	1 (4.3)	6 (3.8)	14 (4.8)	0.608
*Klebsiella pneumoniae (n = 24)*	5 (5.7)	-	4 (2.6)	15 (5.1)	0.407
*Corynebacterium* spp. *(n = 17)*	4 (4.5)	1 (4.3)	4 (2.6)	9 (3.1)	0.844
*Strep. Alpha hemolytic (n = 11)*	-	1 (4.3)	4 (2.6)	6 (2.1)	0.731
*Micrococcus luteus (n = 8)*	-	-	5 (3.2)	3(1.0)	0.105
*Strep. Beta hemolytic (n = 8)*	-	-	2 (1.3)	6 (2.1)	0.559
*Salmonella* spp. *(n = 5)*	-	-	2 (1.3)	3 (1.0)	0.811
*Mannheimia haemolytica* *(n = 5)*	-	-	1 (0.6)	4 (1.4)	0.486
*Campylobacter (n = 5)*	1 (1.1)	-	1 (0.6)	3 (1.0)	0.900
*Serratia marcescens (n = 4)*	-		2 (1.3)	2 (0.7)	0.528
*Listeria monocytogenes (n = 2)*	-	-	2 (0.7)	-	-
*Pasteurella multocida (n = 1)*		-	1 (0.3)	-	-

^1^-Proportion of each isolate to total isolated bacteria in the same group. Different superscripts indicate significant difference in rows (*p* < 0.05).

**Table 3:** Data composition of clinical features and frequency distribution of negative and positive culture of uterine samples in the experimental subjects.

	N Cases	+ Culture (%)	− Culture (%)	*p* Value
Animal type				
Sub-fertile ^a^	72	48 (66.6)	24 (33.3)	0.001
Calved ^b^	264	112 (42.4)	152 (57.6)	
Unknown ^b^	476	219 (46.0)	257 (54.0)	
BCS				
≤2	194	95 (48.9)	99 (51.1)	0.112
2–3	357	170 (47.6)	187 (52.4)	
3–4	185	79 (42.7)	106 (57.3)	
4–5	76	31 (40.8)	45 (59.2)	
Age group				
≤10 years	631	290 (45.9)	341 (55.8)	0.248
>10 years	181	89 (49.2)	92 (50.8)	
Ovarian status				
Active	578	267 (46.2)	311 (53.8)	0.361
Inactive	234	112 (47.8)	122 (52.1)	
Uterine discharge				
No discharge (ND) ^a^	407	213 (52.3)	194 (47.6)	0.000
Gelatinous discharge (GD) ^ab^	153	75 (49.0)	78 (50.1)	
Purulent discharge grade 1 (PDG1) ^b^	124	45 (36.3)	79 (63.7)	
Purulent discharge grade 2 (PDG2) ^b^	72	27 (37.5)	45 (62.5)	
Purulent discharge grade 3 (PDG3) ^b^	56	19 (33.9)	37 (66.1)	
Uterine ultrasonography				
No echogenicity in lumen (NE) ^a^	611	265 (43.4)	346 (56.6)	0.000
Moderate echogenic line (MEL) ^a^	109	49 (44.9)	60 (55.1)	
Continuous echogenic line/fluid (CEL) ^b^	92	65 (70.6)	27 (29.4)	

Different superscripts indicate significant difference in the distribution frequency of subjects within groups (*p* < 0.05).

## Data Availability

Not applicable.
